# Phenotypic Profile of Waldenström Macroglobulinaemia B‐Cells: Establishment of a Diagnosis Scoring System and Clinico‐Biological Correlations

**DOI:** 10.1111/jcmm.70620

**Published:** 2025-05-23

**Authors:** Elise Sourdeau, Clémentine Boccon‐Gibod, Aurélien Corneau, Myrto Costopoulos, Clotilde Bravetti, Marine Armand, Elise Chapiro, Florence Nguyen‐Khac, Frédéric Davi, Catherine Blanc, Véronique Leblond, Marine Baron, Damien Roos‐Weil, Magali Le Garff‐Tavernier

**Affiliations:** ^1^ Sorbonne Université, Service d'Hématologie Biologique, Hôpital Pitié‐Salpêtrière APHP Paris France; ^2^ Centre de Recherche des Cordeliers Sorbonne Université, Université Paris Cité, Inserm UMRS 1138, Drug Resistance in Hematological Malignancies Team Paris France; ^3^ Sorbonne Université, Service d'Hématologie Clinique, Hôpital Pitié‐Salpêtrière APHP Paris France; ^4^ Sorbonne Université, INSERM UMS037 PASS, Plateforme de Cytométrie (CyPS) Paris France

**Keywords:** CD38, *CXCR4*, phenotypic scoring system, splenic marginal zone lymphoma, Waldenström Macroglobulinaemia

## Abstract

Waldenström Macroglobulinaemia (WM) is sometimes difficult to differentiate from marginal zone lymphoma (MZL), two entities with overlapping features for which no single marker assessed by multiparameter flow cytometry (MFC) is specific. The aim of this work was to establish a diagnostic phenotypic score for bone marrow (BM) and peripheral blood (PB) WM samples, to differentiate it from MZL and to improve the detection of small circulating WM clones. This study revealed a distinct phenotypic profile between WM and MZL B‐cells. WM B‐cells showed decreased expression of CD19, FMC7, CD22, CD27 and increased expression of CD79b and CD13. Supervised and unsupervised MFC analyses were used to define a phenotypic scoring system: a score of 3/6 or greater in BM or 4/7 or greater in PB samples supported the diagnosis of WM (sensitivity of 97.9% and 94.1% and specificity of 80.0% and 93.5%, respectively). These results were validated in a prospective cohort with very high sensitivity and specificity for the two scoring systems. Clinico‐biological correlations showed that the absence or low expression of CD38 on BM WM B‐cells was significantly associated with increased BM and PB infiltration (*p* < 0.0001 and *p* = 0.0024 respectively) and *CXCR4* mutation (*p* < 0.0001). These results demonstrate that MFC can be used to differentiate WM from MZL with a scoring system that can be easily implemented in routine practice.

## 1 Introduction

Waldenström Macroglobulinaemia (WM) is a rare chronic B‐cell neoplasm. It is characterised by bone marrow (BM) involvement with a tumoral population composed of a continuum of small lymphocytes (Ly), plasmacytoid lymphocytes and plasma cells. These tumour cells secrete a serum monoclonal immunoglobulin M (IgM) that accumulates in the peripheral blood (PB) [1]. The clinical and biological presentation of WM patients is heterogeneous, and differential diagnosis with other B‐cell neoplasms can be challenging, particularly with marginal zone lymphoma (MZL). Indeed, monoclonal IgM component detection is common in MZL, and the *MYD88* L265P mutation, which is found in over 90% of WM patients, can also be detected in some MZL cases [2, 3]. Conversely, spleen enlargement, which is more commonly associated with MZL, is sometimes observed in WM patients. Therefore, to achieve precise diagnostic characterisation, it is necessary to integrate multiple laboratory approaches, including multiparameter flow cytometry (MFC) in BM and PB. The commonly described phenotype of WM B‐cells is IgM^+^, CD19^+^, CD20^+^, CD5^±^, CD10^±^, CD23^±^, CD22^+^, FMC7^+^, CD25^+^, CD27^+^ and CD103^−^, often accompanied by a CD38^+^/CD138^+^ plasma cell component [4]. However, the expression of either of these markers is not constant. Therefore, no single marker can confirm or exclude a diagnosis of WM or distinguish it specifically enough from MZL. Although a combination of different MFC marker expressions can be very suggestive of WM diagnosis [5, 6, 7], no precise immunophenotypic scoring system, such as the one that exists for chronic lymphocytic leukaemia, is available for WM [6, 8, 9, 10].

Therefore, we aimed in a large cohort of WM patients to (i) better define the MFC profile of BM WM B‐cells and (ii) to compare it to immunophenotypic characteristics of MZL B‐cells in order to design a diagnostic scoring system to better discriminate these two entities. Additionally, we improved the detection of circulating tumour WM B‐cells through this phenotypic study. Finally, we correlated the BM phenotype of WM B‐cells with clinical and biological features.

## 2 Methods

### 2.1 Patients

Patients diagnosed with WM (*n* = 133) or MZL (*n* = 56) at Pitié‐Salpêtrière Hospital (Paris, France) were retrospectively included based on medical records and the availability of BM and/or PB immunophenotypic analysis. For WM cases, the diagnosis, treatment initiation and response criteria followed the WHO classification and recommendations from the tenth International Workshop for Waldenström's Macroglobulinaemia (IWWM) [11]. This included a systematic BM biopsy at diagnosis. MZL cases consisted of 27 splenic, 28 nodal and one extranodal case. All cases were confirmed histologically by biopsy of either BM, spleen or lymph nodes. We also incorporated 11 MFC analyses from healthy controls, with no prior diagnosis of gammopathy or haematological disease, including normal BM (*n* = 2) or PB (*n* = 9) samples. In total, 55 patients (29 WM and 26 MZL) diagnosed in 2023 and 2024 were then included in a prospective validation cohort. Written consents were obtained in accordance with the declaration of Helsinki and with ethical approval from national (CNIL 2212382) and local (CPP Ile‐De‐France 05/21/2014) ethics committees.

### 2.2 Flow Cytometry Analyses

MFC analyses were performed on erythrocyte‐lysed BM or blood samples between 2015 and 2024 with the same MFC panel. A seven‐colour/three‐tube panel was used for B lymphocyte phenotype assessment: (i) kappa(κ)/lambda(λ) light chain‐FITC/PE, CD19‐PC5.5, CD5‐PE‐Cy7, CD79b‐APC, CD20‐PB and CD45‐HV500; (ii) FMC7‐FITC, CD38‐PE, CD19‐PC5.5, CD5‐PE‐Cy7, CD23‐APC, CD43‐APC‐AF750 and CD45‐HV500; (iii) κ/λ light chain‐FITC/PE, CD19‐PC5.5, CD13‐PE‐Cy7, CD22‐APC, CD27‐BV421 and CD45‐HV500 (Table S1). Data were acquired on a standardised FACS Canto II (BD Biosciences) cytometer with a minimal number of 150,000 leukocytes registered, processed on FACS Diva (BD Biosciences) software and analysed using Kaluza software (Beckman Coulter). The percentage and mean fluorescence intensity (MFI) of each marker on clonal and normal residual B‐cells (for patients) and normal B‐cells (for control subjects) have been recovered. The threshold of 30% was chosen to consider a B‐cells antigen positivity expression, except for CD13 for which the threshold was set at 2% as previously published [10]. The flow cytometry analysis strategy is described on Figure S1. Analyses were performed by quadrants to distinguish positive and negative markers (for the flow cytometry profiles, first part of the results). However, we evaluate clonal B‐cells and marker positivity in the entire pathological B‐cell population when considering the scoring systems. Unsupervised MFC analyses were performed using Omiq and R software (version 4.0.3). Principal component analysis (PCA) was performed on the 86 BM WM samples used for immunophenotypic analysis. The results demonstrated the absence of a batch effect despite the analysis of samples collected across several years (Figure S2), thereby facilitating the comparison of results across different time points.

### 2.3 Cytogenetic and Molecular Analyses

Chromosome banding analyses were performed according to usual techniques [12]. All karyotypes were described according to the International System for Human Cytogenetic Nomenclature (ISCN 2020). FISH was performed on interphase nuclei and metaphases, following standard procedures and using specific probes: ATM(11q22), TP53(17p13.1), BCL2(18q21), D12Z1(12p11‐q11), D13S319(13q14), LAMP1(13q34), D6Z1(6p11‐q11)/SEC63(6q21)/MYB(6q23) and FIP1L1/CHIC2/PDGFRA(4q12) deletion/fusion.

Genomic DNA was extracted using the QiAamp DNA Micro Kit (Qiagen) from non‐sorted BM samples. Assessment of *MYD88* and *CXCR4* mutations was performed by either restriction fragment length polymorphism, allele‐specific polymerase chain reaction or next generation sequencing [13].

### 2.4 Statistical Analyses

All statistical analyses were performed with appropriate tests using GraphPad Prism 9 (version 9.0.0) or STATA (version 14.0) software. Phenotypic characteristics were compared using Mann–Whitney tests and correlations assessed with the Spearman test. Multivariate analyses were performed by logistic regression. Performance of BM and PB scoring systems has been determined by using nonparametric ROC curves, with area under the curve (AUC), sensitivity (Se), specificity (Sp), positive predictive value (PPV) and negative predictive value (NPV). The optimal decision threshold was chosen by the maximum of the Youden index. Variable importance analysis was performed to rank variables by predictive power according to the Random Forest algorithm. Clinical and phenotypical correlations were realised for 114 WM patients with GraphPad Prism and STATA software. *p*‐values below 0.05 were considered statistically significant (*p ≤ 0.05; **p ≤ 0.01; ***p ≤ 0.001 and ****p ≤ 0.0001).

## 3 Results

### 3.1 Flow Cytometry Profile of BM WM B‐Cells

**FIGURE 1:**
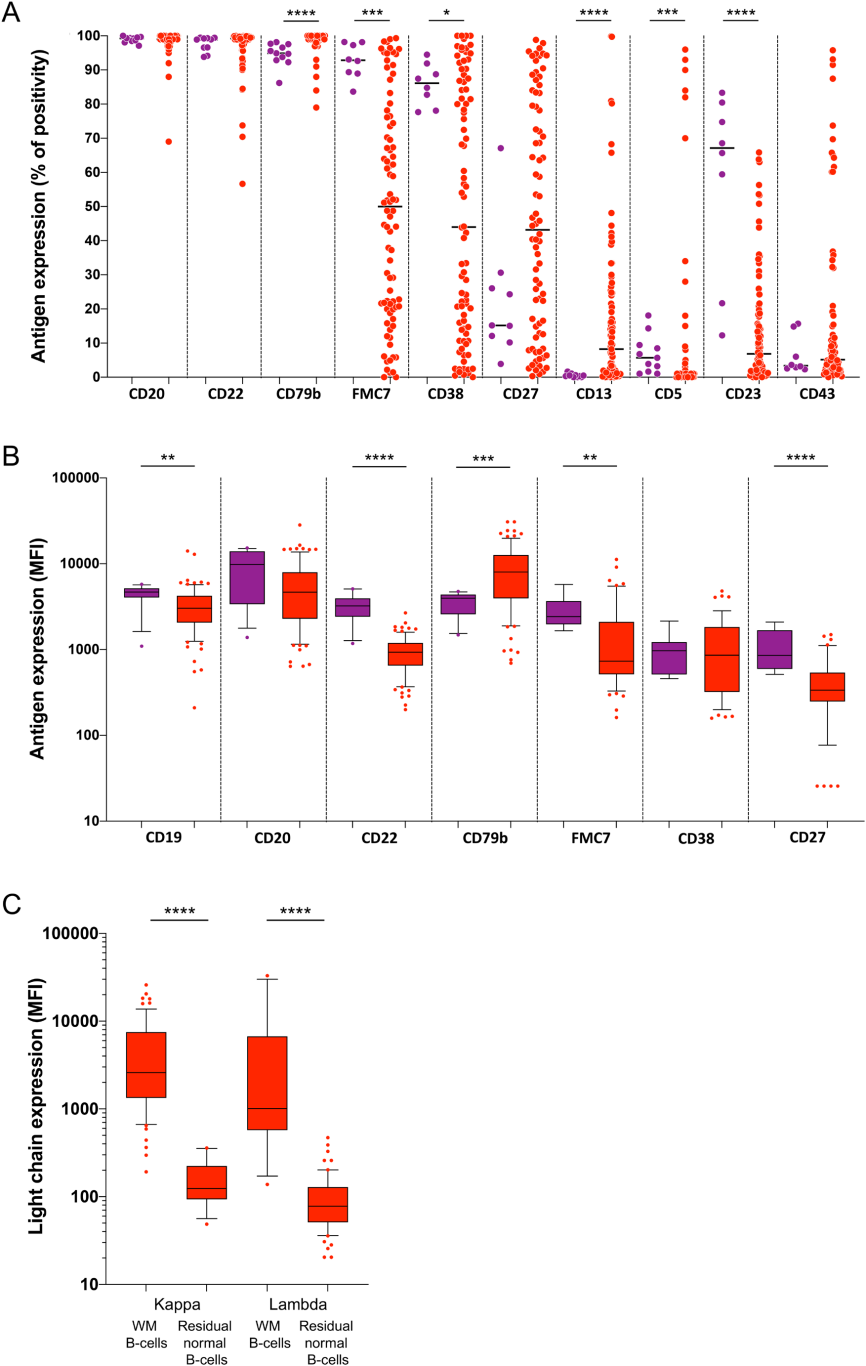
Comparison of antigen expression on B‐cells between healthy controls (*n* = 11) and WM patients (*n* = 86). (A) Repartition of percentage antigen expression on normal B‐cells (control subjects) compared to clonal BM WM B‐cells (WM patients). For each marker, data are represented with control subjects on the left (violet points) and WM patients on the right (red points). (B) MFI of antigen expression on normal B‐cells (control subjects) compared to clonal BM WM B‐cells (WM patients). For each marker, boxplot with the median and the interquartile range of MFI are represented with control subjects on the left (violet points) and WM patients on the right (red points). (C) Boxplot with the median and the interquartile range of MFI of light chain restriction expression on clonal BM WM B‐cells compared to residual normal B‐cells. *p ≤ 0.05; **p ≤ 0.01; ***p ≤ 0.001 and ****p ≤ 0.0001.

We first evaluated the BM MFC analyses of 86 WM patients to define their immunophenotypic profiles more accurately. The mean BM clonal B‐cell infiltration, assessed by MFC, was 45% (range, 2–92) of total lymphocytes. As expected, clonal B‐cells from WM patients consistently expressed the pan‐B markers CD19, CD20, CD22 and CD79b and a restricted light chain with a predominance of κ (79% of patients) over λ cases. CD5, CD23 and CD43 were mostly negative, found in 9%, 18% and 22% of patients, respectively. The expression of FMC7, CD38 and CD27 was widely heterogeneous among patient samples. Finally, CD13 was expressed in two‐thirds of patients when applying the previously described 2% threshold of positivity (Figure 1A). Then, we assessed antigen expression modulations by comparing MFI data of clonal B‐cells from WM patients with those of mature B‐cells from healthy controls (Figure 1B, Table S2), for all positive markers, except CD13, which is not expressed on normal B‐cells. In comparison to healthy controls, WM B‐cells exhibited stronger expression of CD79b (*p* = 0.001), while consistently lower expression of CD19, CD22, FMC7 and CD27 in WM was observed (*p* = 0.0089, *p* < 0.0001, *p* = 0.0035 and *p* < 0.0001, respectively) (Table S3). There was no significant difference in CD20 and CD38 MFI expression. Light chain MFI expression was evaluated by comparing WM B‐cells to residual paired normal B‐cells when available. WM B‐cells expressed strongly either κ or λ light chain (*p* < 0.0001 and *p* < 0.0001, respectively) (Figure 1C), consistent with increased expression of co‐receptor CD79b. The most common immunophenotypic expression profile of WM B‐cells can be summarised as follows: κ or λ strong light‐chain restriction, CD19/CD22^+weak^, CD20^+^, CD79b^+strong^, FMC7/CD27^±weak^, CD13/CD38^±^ and CD5/CD23/CD43^−^. However, some markers, namely FMC7, CD38, CD27 and CD13, showed heterogeneous expression on WM B‐cells.

### 3.2 Comparison of Flow Cytometry Profiles Between WM and MZL B‐Cells

**FIGURE 2:**
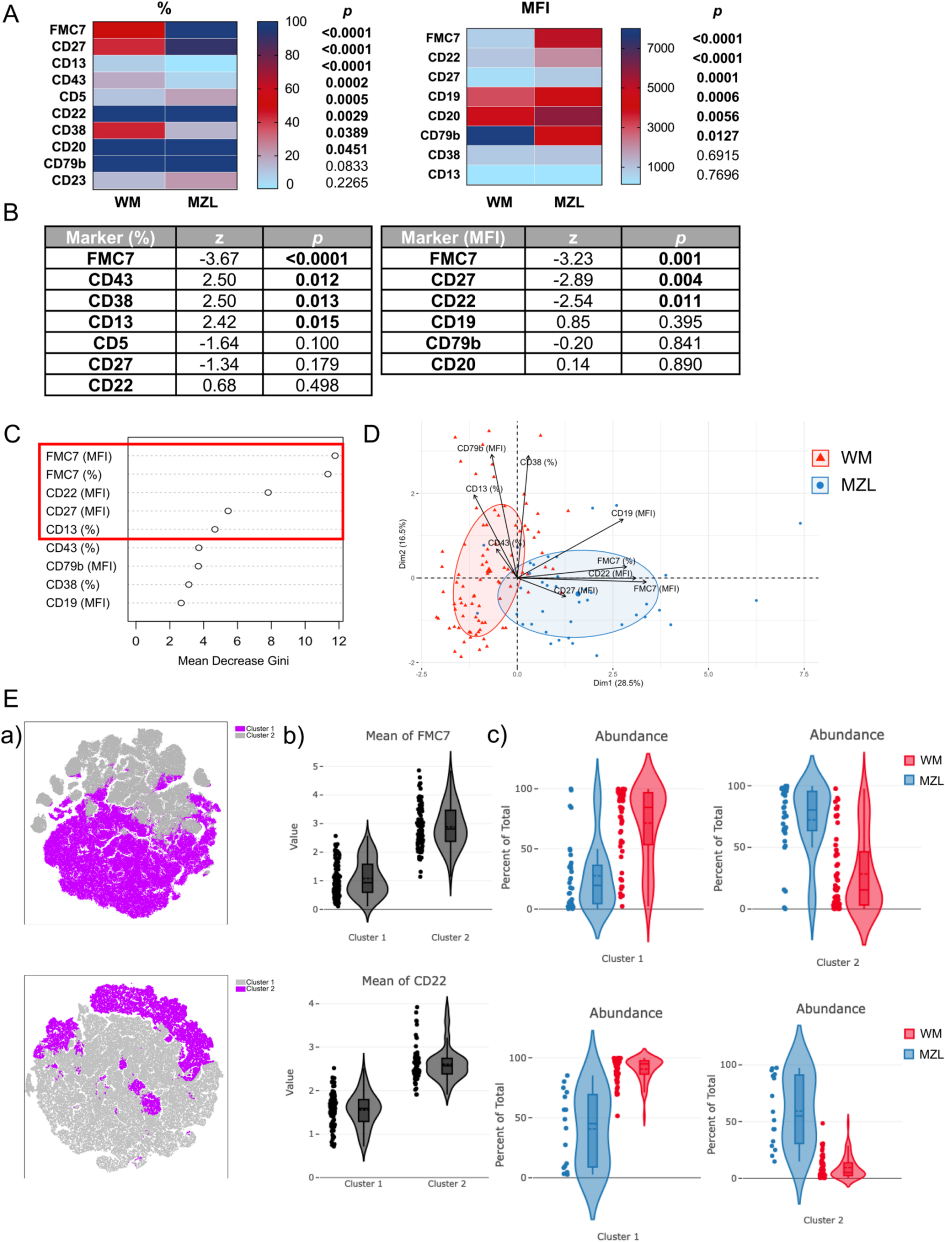
Comparison of antigen expression on WM patients (*n* = 86) and MZL patients (*n* = 40). (A) Heatmap of median phenotypic markers expression (percentages and MFI) with the corresponding *p*‐values. (B) Logistic regressions on significant univariate analysis markers. *z* corresponding to the regression coefficient. (C) Performance of the predicted model by the Random Forest algorithm. Representation of the importance of each variable in model prediction according to the Gini index. The top four parameters ranked are based on the mean decrease in Gini coefficient (a measure of homogeneity from 0 [homogeneous] to 1 [heterogeneous]). (D) PCA on phenotypic significant results for CD43, CD38, FMC7 and CD13 percentages and CD19, CD79b, FMC7, CD27 and CD22 MFI between WM patients (red triangles) and MZL patients (blue points). The two largest triangle and point represent the centers of gravity of each patients group. Concentration ellipse contains 50% of the individuals. (E) (a) t‐SNE analysis of CD19^+^ cells. Cells are automatically separated into spatially distinct subsets according to the combination of markers that they express. t‐Mapping were generated using all markers. The settings run were: Iterations (2000), perplexity (50) and theta (0.5). The upper t‐SNE corresponds to panel with FMC7, CD38, CD19, CD5, CD23, CD43 and CD45 (tube 2), the lower t‐SNE corresponds to panel with CD19, CD5, CD22, CD27, CD45, CD13, kappa/lambda light chain (tube 3). The t‐SNE maps are presented based on 2 clusters: For tube 2, cluster 1 does not express the FMC7 marker, while cluster 2 does; for tube 3, cluster 1 does not express the CD22 marker, while cluster 2 does. (b) Violin plot showing the MFI of FMC7 for tube 2 (top row) and the MFI of CD22 for tube 3 (bottom row) based on the 2 clusters. (c) Proportion of clusters based on MZL and WM for tube 2 (top row) and for tube 3 (bottom row).

To improve differential diagnosis with MZL, we then compared MFC data from 40 MZL samples to those of the 86 BM WM samples described above. No differences were observed between WM and MZL cells regarding κ versus λ light chain restriction (5:1) and the strong expression of light chain expression. Univariate analyses revealed differential expression of several surface antigens between WM and MZL, including CD19, CD20, CD79b, CD5, CD43, CD38, FMC7, CD22, CD27 and CD13 (Figure 2A). On a biparametric dot‐plot, we noticed a differential FMC7/CD38 B‐cell distribution between WM and MZL with a trend towards overexpression of FMC7 and underexpression of CD38 on MZL B‐cells (Figure S3A). A logistic regression was performed on significant univariate analysis markers to identify the most relevant antigens for discriminating between WM and MZL. The percentage expressions of CD43 (*p* = 0.012), CD38 (*p* = 0.013) and CD13 (*p* = 0.015) were found to be higher in WM than in MZL, while FMC7 expression (*p* < 0.0001) was significantly lower in the WM group. Additionally, the MFI of CD22 (*p* = 0.011) and CD27 (*p* = 0.004) were significantly weaker in WM than in MZL clonal B‐cells (Figure 2B). A random forest model and a PCA confirmed these data with a discriminant role for the four parameters FMC7, CD22, CD27 and CD13 (Figure 2C,D). The predictive algorithm correctly classified 83 out of 86 (97%) patients in the WM group and 33 out of 40 (83%) patients in the MZL group based on the selected markers, including percentages of CD43, FMC7, CD38, CD13, and MFI of CD19, FMC7, CD22, CD27 and CD79b. Finally, we analysed immunophenotypic data from 56 WM patients and 34 MZL patients using Omiq software. This unsupervised MFC analysis involved dimensionality reduction (using t‐SNE) and clustering (using FlowSOM). The results confirmed our previous findings of a distinct profile between WM and MZL (Figure 2E, Figure S4). Once again, the most significant differences were observed in the fluorescence intensity of FMC7 and CD22 antigens, which were higher in the MZL group than in the WM group.

### 3.3 WM/MZL and MFC Scoring Systems

**FIGURE 3:**
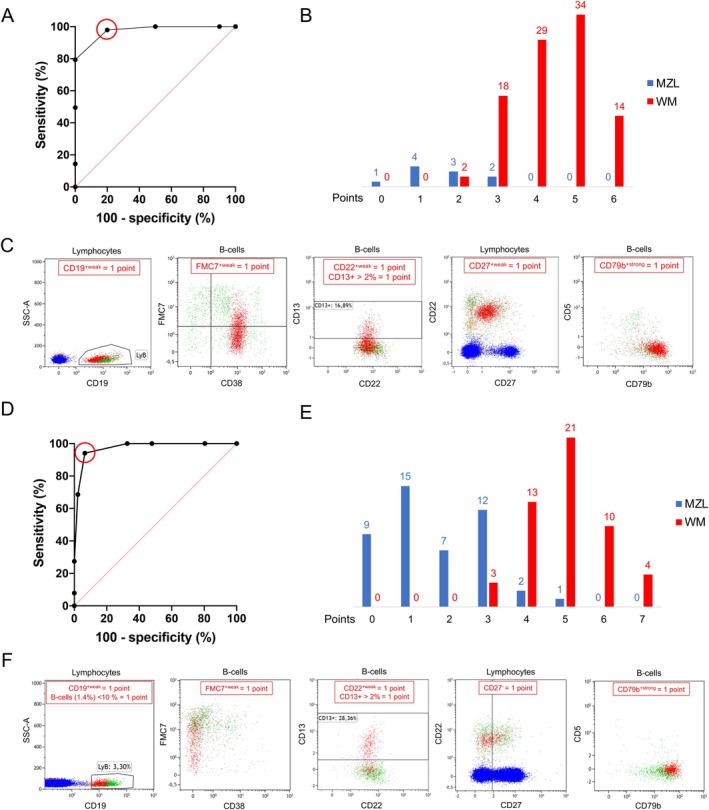
Performances of the BM and PB WM/MZL scoring systems. (A) ROC curve with Se and 100‐Sp according to results of BM score. (B) Number of WM and MZL patients according to each result of BM‐based score. (C) Application of the marrow‐based score on a WM patient's BM sample. Biparametric dot‐plots useful to determinate result of the score: WM B‐cells are in red, residual B‐cells are in green and T‐cells in blue. (D) ROC curve with Se and 100‐Sp according to results of PB‐based score. (E) Number of WM and MZL patients according to each result of PB‐based score. (F) Application of the PB score on a WM patient's blood sample. Biparametric dot‐plots useful to determinate result of the score: WM B‐cells are in red, residual B‐cells are in green and T‐cells in blue.

We have developed a WM BM‐based immunophenotypic scoring system to differentiate WM from MZL. The system is based on the six most relevant parameters (Table 1), including FMC7, CD22, CD27 intensity expression and CD13 percentage. Additionally, CD79b MFI was also included due to its importance in highlighting WM cells in association with CD22. The CD79b/CD22 MFI ratio was significantly higher on WM than MZL B‐cells (8.3 vs. 1.6, *p* < 0.0001) (Figure S3B). The final parameter, CD19, was useful in distinguishing WM and MZL patients due to its weaker expression in WM (*p* = 0.0006). One point was given for each marker except for CD79b, for which one point was given only for strong CD79b expression in the case of CD22^+weak^. We chose not to include CD38 in our scoring system due to its highly heterogeneous expression among WM patients. The decision threshold for this score was determined on 97 WM patients and 10 MZL patients. ROC curve (AUC = 0.9742) indicated that a score greater than or equal to 3/6 was in favour of WM with the best Se (97.9%) and Sp (80.0%) (Figure 3A). Similarly, PPV was equal to 97.9% and NPV to 80.0%. Only two WM patients had a score equal to 2 points (Figure 3B). All except two patients with MZL had a score equal to less than 3 points. These two MZL patients had a strong expression of FMC7, which supports this diagnosis. Figure 3C illustrates the application of the score to a 6‐point BM sample from a patient with WM.

**TABLE 1:** Bone marrow WM/MZL scoring system based on six parameters.

	0 point	1 point
CD19 expression	Normal or strong	Weak
FMC7 expression	Normal or strong	Negative or weak
CD22 expression	Normal or strong	Weak
CD79b expression if CD22^−/weak^	Weak or normal	Strong
CD27 expression	Normal	Negative or weak
CD13 percentage	< 2%	≥ 2%

Before transposing this BM based score to the PB compartment, we compared BM with PB MFC analyses for 15 WM patients, for whom paired samples were available (Bland–Altman test, data not shown). The expression of FMC7 was significantly higher on PB cells than on BM cells (*p* = 0.0320). However, when comparing PB FMC7 expression (percentage and MFI) on WM and MZL cells, this marker remained significantly decreased in WM compared to MZL (*p* = 0.0007 and *p* = 0.0036, respectively).

The WM blood MFC scoring system was therefore based on the same six parameters as the BM score, with the addition of the percentage of clonal B‐cells in the total lymphocytes count (Table 2). This parameter was significantly lower in WM patients compared to MZL patients (median, 7% vs. 52%; *p* < 0.0001). To facilitate its use, a threshold of 10% has been chosen. The PB score decision threshold was determined among 51 WM patients and 46 MZL patients. The ROC curve (AUC to 0.9729) indicated that a score equal to or greater than 4/7 favours WM with the best Se (94.1%) and Sp (93.5%) (Figure 3D). Similarly, the PPV was equal to 94.1% and NPV to 93.5%. Only two MZL patients had a cutoff score of 4 points and one patient had a score of 5 points, with a strong expression of FMC7 supporting this diagnosis for two of them. Only three WM patients had a score of 3 points (Figure 3E). Figure 3F presents the application of the PB score on a PB sample from a patient with WM, resulting in a score of 7 points.

**TABLE 2:** Peripheral blood WM/MZL scoring system based on seven parameters.

	0 point	1 point
Clonal B cells among total lymphocytes percentage	≥ 10%	< 10%
CD19 expression	Normal or strong	Weak
FMC7 expression	Normal or strong	Negative or weak
CD22 expression	Normal or strong	Weak
CD79b expression if CD22^−/weak^	Weak or normal	Strong
CD27 expression	Normal	Negative or weak
CD13 percentage	< 2%	≥ 2%

The markers used in the PB‐based score were helpful in distinguishing and isolating WM B‐cells from normal B‐cells. This was particularly useful when circulating tumour cells (CTCs) were present in very low amounts, or when mixed with a majority of normal residual B‐cells. The CD79b/CD19 biparametric dot‐plot was especially important in this regard.

A prospective validation cohort was assembled from samples obtained from 29 WM and 26 MZL patients diagnosed in 2023 and 2024. The application of the BM MFC scoring system to 22 WM and 8 MZL samples demonstrated a sensitivity of 100% and a specificity of 87.5%. Similarly, the application of the blood MFC scoring system to 13 WM and 22 MZL samples exhibited a sensitivity of 92.3% and a specificity of 95.5%.

### 3.4 MFC, Clinical and Biological Correlations in WM

**FIGURE 4:**
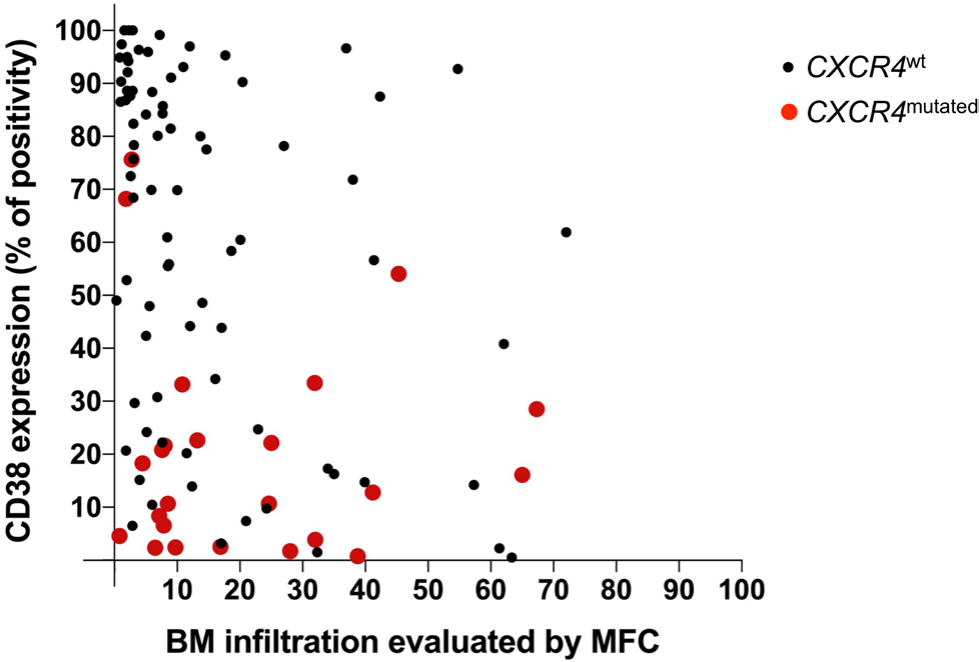
CD38 expression according to WM B‐cells BM infiltration evaluated by MFC and *CXCR4* mutational status (*n* = 74). Patients with a mutation of *CXCR4* are represented with a larger diameter red dot than patients unmutated for *CXCR4* who are represented by a small black dot.

Finally, we analysed the correlation between main clinico‐biological features and phenotypic characteristics in the entire WM cohort. In both univariate and multivariate analyses, no single studied antigen was significantly associated with hyperviscosity syndrome, adenopathy, anaemia, serum IgM level, elevated β2‐microglobulin, cytogenetic abnormalities, *MYD88* mutation, number of lines of treatment, international prognostic scoring system for WM, treatment response, progression‐free survival or overall survival (data not shown). However, the absence or low expression of CD38 was significantly correlated with splenomegaly (*p* = 0.0444), high level of lactate dehydrogenase (*p* = 0.01), initiation of a treatment (*p* = 0.0325), a decreased platelet count (*p* = 0.0063), an increased BM (*p* < 0.0001) (Figure 4) and blood tumoral infiltration (*p* = 0.0019) and the presence of a *CXCR4* mutation (S338 and others) (*p* < 0.0001) (Figure 4).

Furthermore, PCA analysis on 74 WM patients with or without *CXCR4* mutation (S338 and others) revealed a distinct MFC profile with a separation into two groups (Figure S5A). Indeed, associated with the diminution of CD38 expression, *CXCR4*‐mutated WM clonal B‐cells demonstrated decreased expression of CD19 (*p* = 0.0002), CD79b (*p* = 0.0076), FMC7 (*p* = 0.0257) and CD27 (*p* = 0.0280) compared to *CXCR4*‐unmutated WM clonal B‐cells (Table S4, Figure S5B–C).

## 4 Discussion

Due to its heterogeneous clinical and biological presentation, WM can be difficult to distinguish from other monoclonal IgM‐secreting entities such as MZL. This study revealed that the most common immunophenotypic expression profile of WM B‐cells is defined by strong light‐chain restriction, CD19/CD22^+weak^, CD20^+^, CD79b^+strong^, FMC7/CD27^±weak^, CD13/CD38^±^, CD5/CD23/CD43^−^. We investigated the potential differences in antigenic expression between WM and MZL cells, two types of lymphoplasmacytic‐differentiated lymphomas. We identified a distinct MFC profile, characterised by low expression of CD19, FMC7, CD22 and CD27 antigens, strong expression of CD79b, and partial positivity for CD13 on WM tumoral B‐cells. These results are consistent with those published by Paiva et al. who used 17 antigenic markers [14]. Our findings enabled the development of two scoring systems for the diagnosis of WM. The BM and PB scores were based on six and seven parameters respectively. In the future, a respective threshold of 3/6 and 4/7 could be used for routine diagnosis. These results were validated in a prospective cohort with very high sensitivity and specificity for the two scoring systems.

Other notable results include the striking association between the presence of a *CXCR4* mutation and the absence or weak CD38 expression on WM B‐cells, which is also in line with previously published data [15]. We have identified differences between mutated and non‐mutated *CXCR4* WM patients with low CD38 expression, as well as lower expression of CD19, CD79b, FMC7 and CD27 markers. The study published in 2016 by Poulain et al. did not describe these phenotypic variations. However, it did reveal lower CD138 expression in the *CXCR4* mutated group (which was not evaluated in the present study), with no difference in CD38 and CD27 expression [16].

Some limitations to our study relate to the changes in the antibodies used in the laboratory over its long timespan, particularly for CD25, whose reactivity varies between clones as well as fluorochromes used (Figure S6). Therefore, although the CD25^+^/CD22^+weak^ association has been described as a phenotypic feature of the WM tumour cell, it could not be assessed in this study. Further validation of our findings will also require a prospective, oligo‐ or multicentre study to reduce variability and ensure robustness. This effort is currently underway in France through a national multicentre study. A multicentric approach would also facilitate harmonisation across different MFC platforms, enabling future automation of multi‐centre data interpretation. Nevertheless, the role of the biologist in interpreting MFC results remains crucial. One of the strengths of our WM scoring system is that it relies on antigen markers that are routinely used and widely available in haematology laboratories performing MFC analysis for B‐cell lymphomas (CD19, CD79b, CD22, FMC7 and CD27) and myeloid neoplasms (CD13). Furthermore, similar multi‐marker scoring systems have already been developed and successfully adopted in clinical practice, notably in CLL [8] and hairy cell leukaemia [17, 18].

Improvements in routine MFC techniques with the use of 12/13‐colour panels could also be beneficial. Thanks to these technical improvements, new cytometers now allow the use of a single tube‐panel containing all the markers of interest in our study, including CD45, CD19, κ, λ, CD79b, CD22, CD27, CD13, CD38, FMC7 and CD25. This leaves one or two fluorescence channels free for other markers, depending on the practice of the centre (such as CD180, CD200, LAIR1) [19] or the sample type (such as the addition of CD138 for plasma cells detection in BM). One particular candidate of interest is CD180, a related member of the toll‐like receptor family, which has been found to be overexpressed at the plasma membrane in circulating cells of MZL [20, 21]. This approach will facilitate the detection of CTCs in PB samples [22] and aid in distinguishing between WM and MZL B‐cells. Finally, our results may have potential therapeutic implications. The arsenal of monoclonal antibodies and bispecifics is constantly expanding in B‐cell malignancies, with increasingly diverse targets such as CD20/CD19/CD22/CD79b. These targets may have different activity on WM or MZL B‐cells, given the different expression levels observed for some of these markers.

In conclusion, this work presents an overview of the most distinguishing phenotypic markers between WM and MZL. It highlights the strong value of MFC in distinguishing these two entities. Two scoring systems have been established, one for BM and one for PB samples, which allow for the correct distinction of WM over MZL with a PPV of over 94%. These scores are based on a limited number of markers, each given one point, making them simple to use and easily implementable in routine diagnostics.

## Author Contributions

**Elise Sourdeau:** conceptualization (lead), data curation (lead), formal analysis (lead), methodology (lead), project administration (lead), validation (lead), writing – original draft (lead). **Clémentine Boccon‐Gibod:** conceptualization (lead), data curation (lead), formal analysis (equal), methodology (lead), project administration (lead), validation (lead), writing – original draft (lead). **Aurélien Corneau:** data curation (equal), formal analysis (equal), writing – original draft (supporting). **Myrto Costopoulos:** data curation (equal), methodology (equal), project administration (equal), writing – original draft (supporting). **Clotilde Bravetti:** data curation (equal), formal analysis (equal), writing – original draft (supporting). **Marine Armand:** data curation (equal), formal analysis (equal), writing – original draft (supporting). **Elise Chapiro:** data curation (equal), formal analysis (equal), writing – original draft (supporting). **Florence Nguyen‐Khac:** data curation (equal), formal analysis (equal), writing – original draft (supporting). **Frédéric Davi:** writing – original draft (supporting). **Catherine Blanc:** writing – original draft (supporting). **Véronique Leblond:** writing – original draft (supporting). **Marine Baron:** conceptualization (lead), data curation (equal), formal analysis (equal), methodology (lead), project administration (lead), validation (lead), writing – original draft (lead). **Damien Roos‐Weil:** conceptualization (lead), data curation (equal), formal analysis (equal), methodology (lead), project administration (lead), validation (lead), writing – original draft (lead). **Magali Le Garff‐Tavernier:** conceptualization (lead), data curation (equal), formal analysis (equal), methodology (lead), project administration (lead), validation (lead), writing – original draft (lead).

## Conflicts of Interest

The authors declare no conflicts of interest.

## Supporting information


Data S1.

Data S2.

## Data Availability

The data that support the findings of this study are available from the corresponding author upon reasonable request.
