# Supplementary material for: Phenotypic Profile of Waldenström Macroglobulinaemia B‐Cells: Establishment of a Diagnosis Scoring System and Clinico‐Biological Correlations
Source: J Cell Mol Med. 2025 May 23;29(10):e70620. doi: 10.1111/jcmm.70620 (PMC12101068; doi:10.1111/jcmm.70620)

SUPPLEMENTARY MATERIAL LEGENDS

Supplementary Figure S1 : Flow cytometry analysis strategy used in the study

Leukocytes are coloured pink, clonal B-cells red, residual B-cells black, T-cells blue and natural killer cells brown. The antigens studied are indicated on the abscissa or ordinate axis of the bi-parametric plots. The population studied is indicated at the top of each quadrant. FSC-W: Forward Scatter-Width; FSC-A: Forward Scatter-Area; SSC-A: Side Scatter-Area.

To detail, after selecting the singlets on the FSC-W/FSC-A graph (1), cellular debris are eliminated on the SSC-A/CD45 representation to isolate leukocytes (2). Lymphocyte cells (Ly) are then isolated from leukocytes using a boolean equation based on their low SSC and high CD45 expression (Ly CD45/SSC) (3) and their small size (Ly SSC/FSC) (4). Within Ly, LyB are isolated from LyT by their CD19 expression (5). This LyB isolation strategy was applied to each of the three tubes. Each tube was then differentiated by the antibodies used to characterise the pathological B-cells population:

- Biparametric dot-plot representations 6 to 9 concern tube #1. The presence of isotypic restriction is assessed by the distribution of light chains kappa and lambda on the surface of B-cells (6). Expression of CD5 (7), CD79b (8) and CD20 (9) is also assessed. CD5 can also be used to isolate T-cells (7).
- Biparametric dot-plot representations 7, 10, 11 and 12 concern tube #2. The CD19/CD5 graph (7) is used to isolate LyT. Light chains are not sought in this tube, which studies the expression of FMC7 (10), CD23 (10), CD38 (11) and CD43 (12).
- Biparametric dot-plot representations 6, 13 and 14 concern tube #3. The presence of isotypic restriction is once again assessed by the distribution of light chains kappa and lambda on the surface of LyB (6). The expression profiles of CD13 (13), CD22 (13) and CD27 (14) were assessed.

Supplementary Figure S2. Evaluation of immunophenotypic variations with years.

A PCA graphical representation of the percentages of CD20, CD79b, CD22 and MFI of CD19, CD20, CD79b and CD22 for 86 bone marrow WM samples. Each patient is identified by a symbol whose shape and colour correspond to the year of sampling. The centre of gravity of each group is represented by a symbol that is larger than the group symbols. Each concentration ellipse contains 50% of the individuals.

Supplementary Figure S3. (A) FMC7/CD38 MFI and (B) CD79b/CD22 MFI on B tumoral cells according to WM and MZL patients.

Supplementary Figure S4. Unsupervised FCM analyses with Omiq software between WM patients (n=56) and MZL patients (n=34). t-SNE density gradient representations for all tested marker according to each tube and t-SNE colored according to markers fluorescence intensity across panel. Red indicates the highest density of expression.

Supplementary Figure S5. **Comparison of antigen expression on WM patients with (*CXCR4*^{mutated}, n=22) or without a mutation of *CXCR4* (*CXCR4*^{wt}, n=52).**

(A) PCA on phenotypic results for CD20, CD5, CD23, CD43, CD38, FMC7, CD27, CD13 percentages and CD19, CD79b, CD22 MFI between *CXCR4*^{wt} (blue points) and *CXCR4*^{mutated} (red triangles) WM patients. The two largest triangle and point represent the centers of gravity of each patients group. Concentration ellipse contains 50% of the individuals. (B) Repartition of CD20, CD5, CD23, CD43, CD38, FMC7, CD27, CD13 antigen percentage expression on WM B-cells between *CXCR4*^{wt} (blue) and *CXCR4*^{mutated} (red) groups. (C) MFI of CD19, CD79b, CD22 antigen expression on WM B-cells between *CXCR4*^{wt} (blue) and *CXCR4*^{mutated} (red) groups. For each cytometry marker, boxplot with the median and the interquartile range of MFI are represented.

Supplementary Figure S6. CD25 expression on B-cells and T-cells from a WM patient according to different fluorochromes, clones and manufacturer: CD25-BV421 (REF 564033, BD Biosciences, clone 2A3), CD25-PE-Cy7 (REF 335824, BD Biosciences, 2A3), CD25-APC (REF 340907, BD Biosciences, 2A3), CD25-PC5.5 (REF B92458, Beckman Coulter, B1.49.9), CD25-PB (REF A82944, Beckman Coulter, B1.49.9).

Supplementary Figure S1

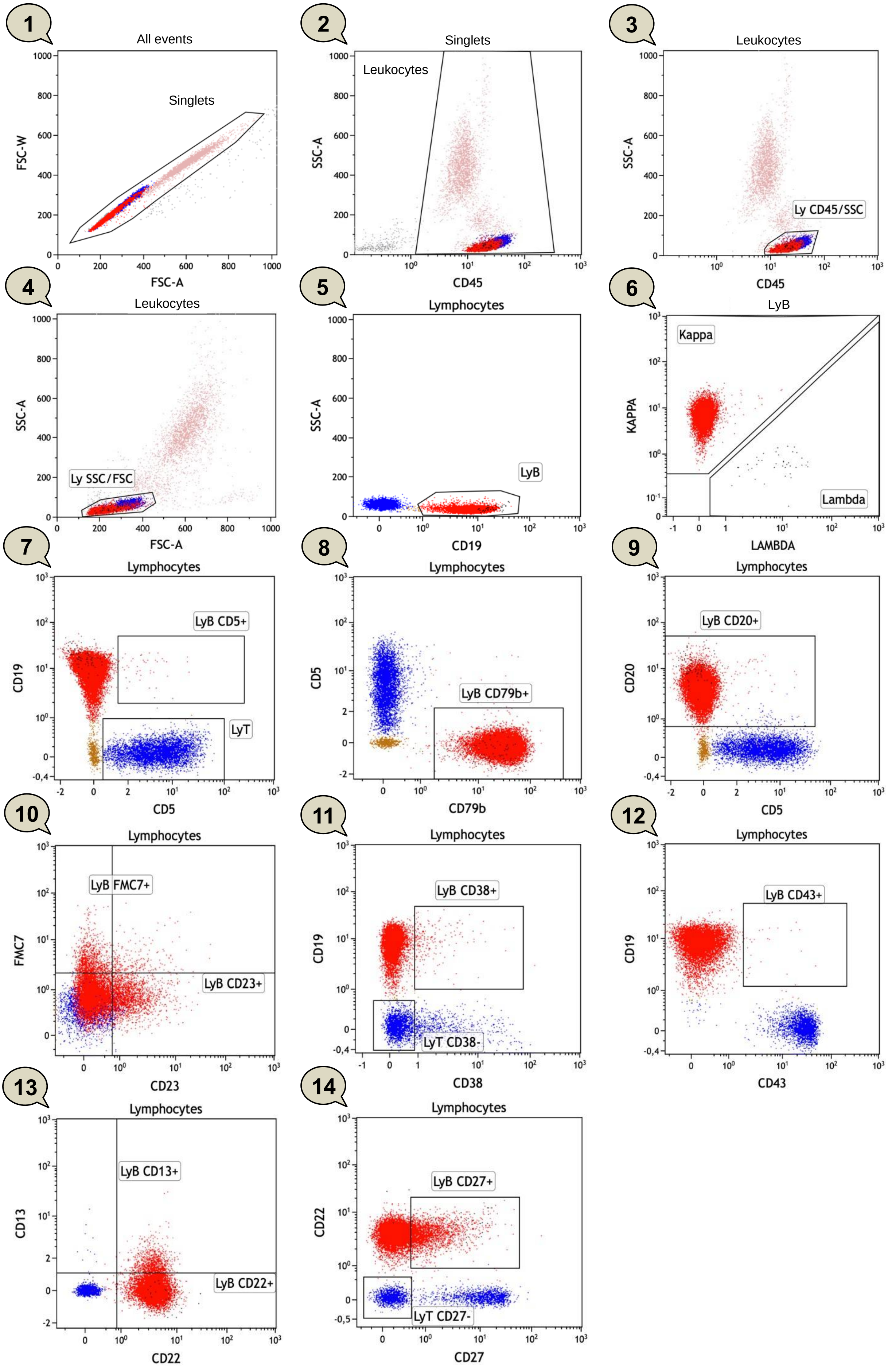

Supplementary Figure S2

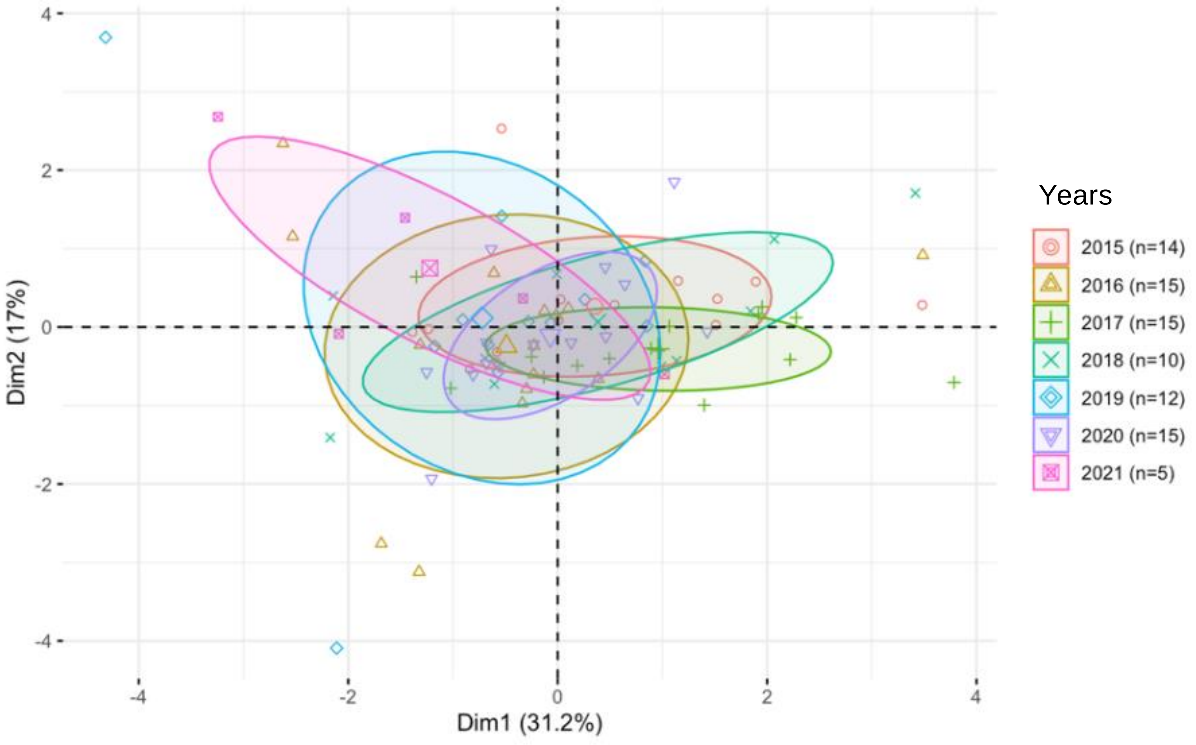

Supplementary Figure S3

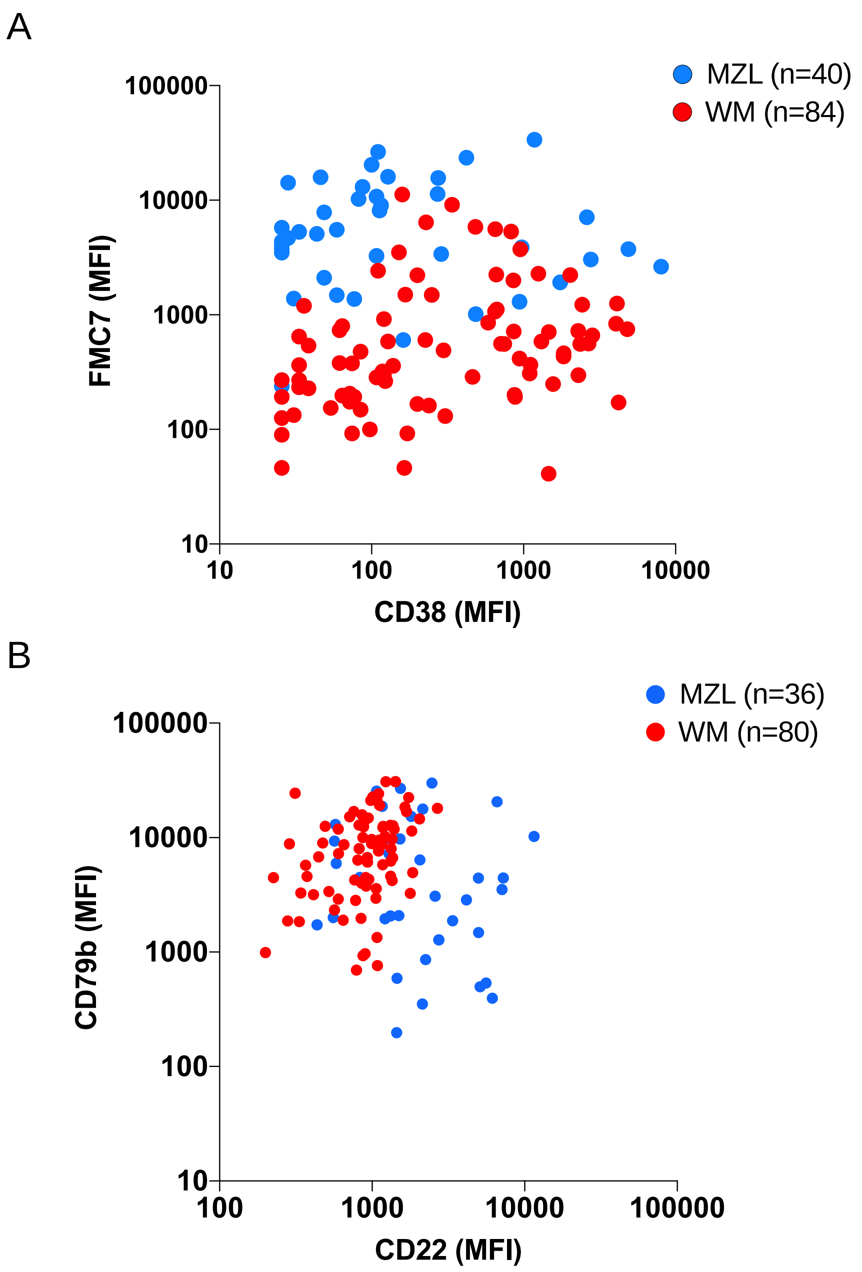

Supplementary Figure S4

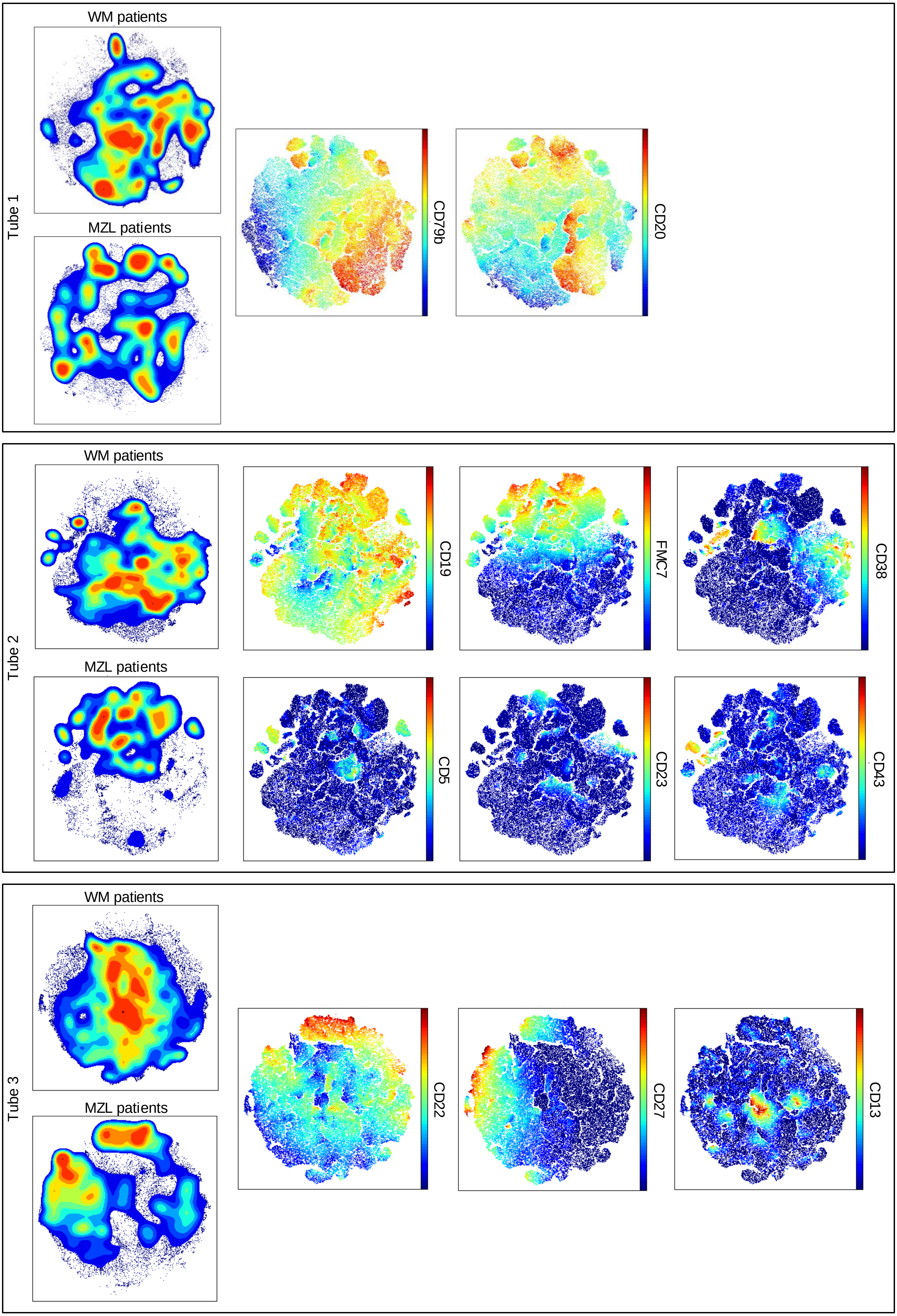

Supplementary Figure S5

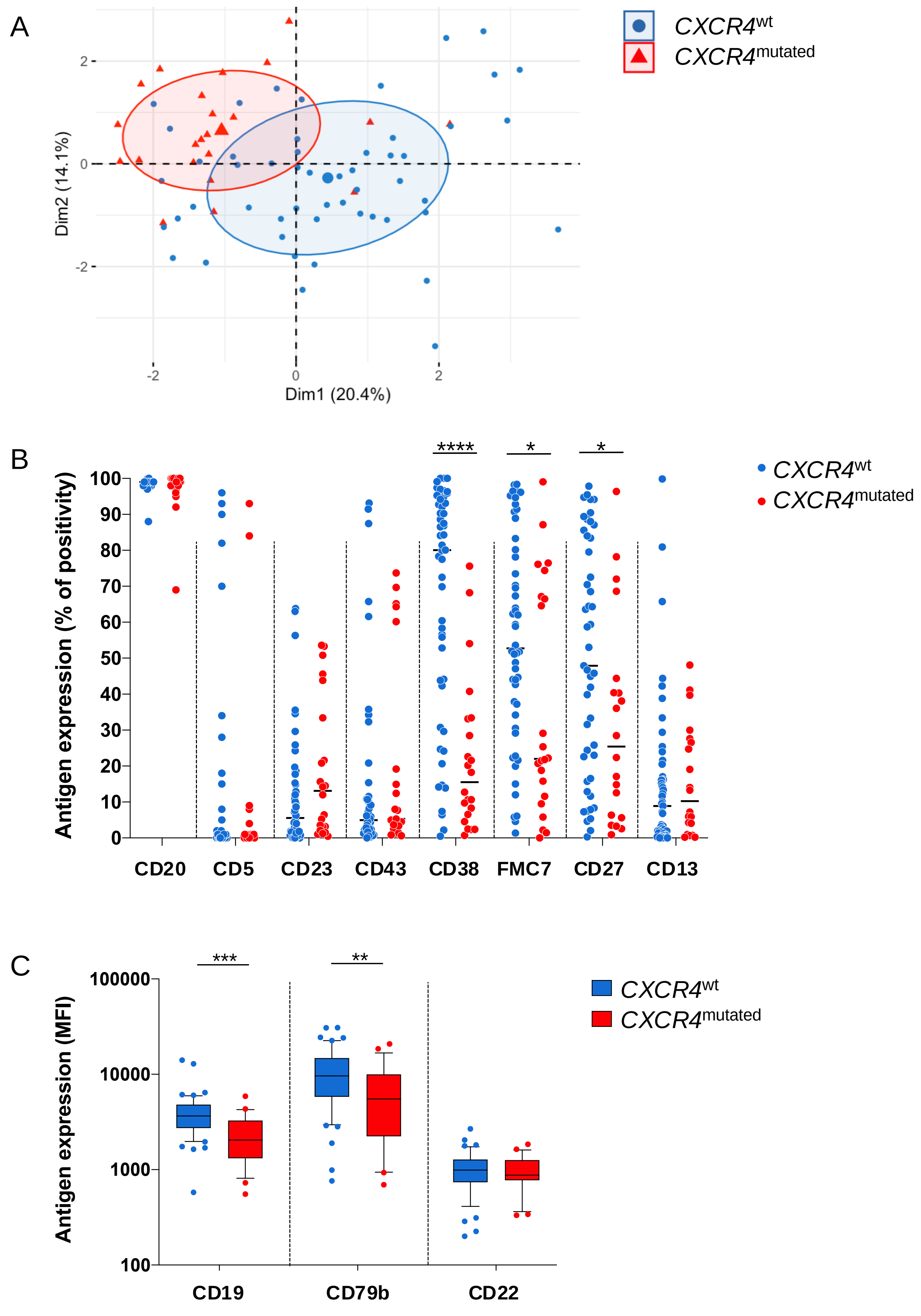

Supplementary Figure S6

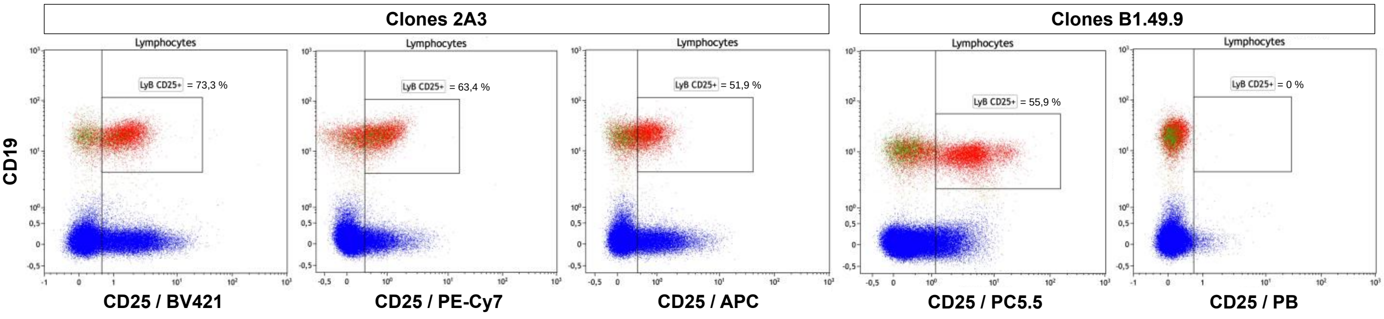

Supplement: Supplementary file 1 — Data S1. [file JCMM-29-e70620-s002.pdf]
