# Supplementary material for: Phenotypic Profile of Waldenström Macroglobulinaemia B‐Cells: Establishment of a Diagnosis Scoring System and Clinico‐Biological Correlations
Source: J Cell Mol Med. 2025 May 23;29(10):e70620. doi: 10.1111/jcmm.70620 (PMC12101068; doi:10.1111/jcmm.70620)
Supplement: Supplementary file 2 — Data S2. [file JCMM-29-e70620-s001.pdf]

SUPPLEMENTARY MATERIAL LEGENDS

Supplementary Table S1. Details of markers with their fluorochrome, clone and manufacturer studied for screening controls, WM and MZL samples. FITC: Fluorescein; PE: Phycoerythrin; PC5.5: Phycoerythrin-Cyanine 5.5; PE-Cy7: PE-Cyanine7; APC: Allophycocyanin; APC-AF750: APC-Alexa Fluor 750; PB: Pacific Blue; BV421: Brilliant Violet 421; HV500: Horizon V500. BD: Beckton Dickinson.

Supplementary Table S2. MFC results for healthy controls, WM patients and MZL patients.
Only MFC data used for firstly comparison of antigen expression on B-cells between healthy controls (2 BM and 9 PB) and WM patients (86 BM) and secondly for comparison of antigen expression on WM patients (86 BM) and MZL patients (6 BM and 34 PB) are represented.

Supplementary Table S3. **Statistical comparison results expressed with p-value between control subjects and WM patients, between WM and MZL patients and between WM B-cells and residual normal B-cells.** Comparisons between WM B-cells and residual normal B-cells were not realized for FMC7, CD38 and CD27 because of heterogeneity expression of these markers and for CD5, CD23 and CD43 because of absence expression of these markers for most of patients.

Supplementary Table S4. **Comparison of characteristics and antigens expression on WM patients with (*CXCR4*^{mutated}, n=22) or without a mutation of *CXCR4* (*CXCR4*^{wt}, n=52).**

Supplementary Table S1

Marker	Fluorochrome	Clone	Manufacturer
Anti-kappa	FITC	Polyclonal	Dako
Anti-lambda	PE	Polyclonal	Dako
CD5	PE-Cy7	L17F12	BD Biosciences
CD13	PE-Cy7	L138	BD Biosciences
CD19	PerCP-Cy5.5	J3-119	Beckman Coulter
CD20	PB	B9E9 (HRC20)	Beckman Coulter
CD22	APC	S-HCL-1	BD Biosciences
CD23	APC	EBVCS-5	BD Biosciences
CD27	BV421	M-T271	BD Biosciences
CD38	PE	HB7	BD Biosciences
CD43	APC-AF750	DFT1	Beckman Coulter
CD45	HV500	HI30	BD Biosciences
CD79b	APC	SN8	BD Biosciences
FMC7	FITC		Beckman Coulter

Supplementary Table S2

		Healthy controls (n=11)	WM patients (n=86)	MZL patients (n=40)
CD19	MFI, median [Interquartile range]	4682 [4047-5179]	3036 [2061-4237]	4433 [2961-6232]
Kappa	MFI, median (n) [Interquartile range)	740 (11) [469-1756]	2591 (68) [1336-7497]	2115 (31) [988-6802]
Lambda	MFI, median (n) [Interquartile range]	405 (11) [289-566]	1009 (14) [572-6707]	1892 (8) [829-4971]
CD20	NA, n	0	1	0
	≥30%, % (n)	100 (11)	100 (85)	100 (40)
	MFI, median [Interquartile range]	9792 [3379-13952]	4657 [2281-7941]	5811 [3640-18563]
	<30%, % (n)	0 (0)	0 (0)	0 (0)
CD22	NA	1	5	3
	≥30%, % (n)	100 (10)	100 (81)	100 (37)
	MFI, median [Interquartile range]	3231 [2414-3949]	932 [649-1196]	2056 [1225-4977]
	<30%, % (n)	0 (0)	0 (0)	0 (0)
CD79b	NA	0	1	0
	≥30%, % (n)	100 (11)	100 (85)	98 (39)
	MFI, median [Interquartile range]	3973 [2570-4370]	7992 [3960-12612]	4419 [1477-9825]
	<30%, % (n)	0 (0)	0 (0)	2 (1)
FMC7	NA	3	2	0
	≥30%, % (n)	100 (8)	63 (53)	98 (39)
	MFI, median [Interquartile range]	2424 [1971-3679]	732 [513-2103]	5076 [3034-11315]
	<30%, % (n)	0 (0)	37 (31)	2 (1)
CD38	NA	3	2	0
	≥30%, % (n)	100 (8)	58 (49)	35 (14)
	MFI, median [Interquartile range]	966 [513-1221]	860 [321-1832]	955 [284-2644]
	<30%, % (n)	0 (0)	42 (35)	65 (26)
CD27	NA	2	8	4
	≥30%, % (n)	22 (2)	60 (47)	89 (32)
	MFI, median [Interquartile range]	855 [595-1683]	335 [248-538]	838 [416-1197]
	< 30%, % (n)	78 (7)	40 (31)	11 (4)
CD13	NA	1	5	2
	≥2%, % (n)	0 (0)	68 (55)	26 (10)
	MFI, median [Interquartile range]	NA	128 [44-238]	136 [49-166]
	<2%, % (n)	100 (10)	32 (26)	74 (28)
CD5	≥30%, % (n)	0 (0)	9 (8)	17 (7)
	<30%, % (n)	100 (11)	91 (78)	83 (33)
CD23	NA	3	2	0
	≥30%, % (n)	75 (6)	18 (15)	33 (13)
	<30%, % (n)	25 (2)	82 (69)	77 (27)
CD43	NA	3	8	2
	≥30%, % (n)	0	22 (17)	3 (1)
	<30%, % (n)	100 (8)	78 (61)	97 (37)

Supplementary Table S3

		WM *vs* healthy controls (*p*)	WM *vs* MZL (*p*)	WM B-cells *vs* residual normal B-cells (*p*)
CD19	MFI, median	0.0089	0.0006	<0.0001
Kappa	MFI, median	0.0003	0.1559	<0.0001
Lambda	MFI, median	0.0051	0.2743	<0.0001
CD20	%, median	0.1867	0.0451	0.0506
	MFI, median	0.0886	0.0056	
CD22	%, median	0.1401	0.0029	<0.0001
	MFI, median	<0.0001	<0.0001	
CD79b	%, median	<0.0001	0.0833	<0.0001
	MFI, median	0.0010	0.0127	
FMC7	%, median	0.0001	<0.0001	NA
	MFI, median	0.0035	<0.0001	
CD38	%, median	0.0196	0.0389	NA
	MFI, median	0.935	0.6915	
CD27	%, median	0.0753	<0.0001	NA
	MFI, median	<0.0001	0.0001	
CD13	%, median	<0.0001	<0.0001	<0.0001
	MFI, median	NA	0.7696	
CD5	%, median	0.0003	0.0005	NA
CD23	%, median	<0.0001	0.2265	NA
CD43	%, median	0.6872	0.0002	NA

Supplementary Table S4

	CXCR4^{wt} (n=52)	*CXCR4*^{mutated} (n=22)	*p*
Hemoglobin (g/dL), median	11.7	10.7	0.0712
Platelets (G/L), median	265	199	**0.0008**
Lymphocytes (G/L), median	1.8	1.7	0.7703
Serum IgM (g/L), median	8.6	21.9	**0.0006**
CD19 (MFI), median [Interquartile range]	3651 [2726-4807]	2043 [1315-3283]	**0.0002**
CD79 (MFI), median [Interquartile range]	9605 [5780-14799]	5513 [2237-9990]	**0.0076**
CD5 (%), median	0.0	0.5	0.9973
CD23 (%), median	7.5	2.8	0.1189
CD43 (%), median	5.0	5.3	0.4943
CD38 (%), median	80.1	15.5	**<0.0001**
FMC7 (%), median	52.8	22.0	**0.0257**
CD22 (MFI), median [Interquartile range]	991 [735-1280]	874 [773-1260]	0.4286
CD27 (%), median	47.9	25.4	**0.0284**
CD13 (%), median	8.9	10.3	0.4421
